# Proper Glyphosate Application at Post-anthesis Lowers Grain Moisture Content at Harvest and Reallocates Non-structural Carbohydrates in Maize

**DOI:** 10.3389/fpls.2020.580883

**Published:** 2020-12-10

**Authors:** Linmao Zhao, Liuyong Xie, Jingli Huang, Yingchun Su, Chunqing Zhang

**Affiliations:** State Key Laboratory of Crop Biology, Agronomy College, Shandong Agricultural University, Tai’an, China

**Keywords:** sugar signaling, carbohydrates, remobilization, pre-harvest desiccation, glyphosate, grain-filling

## Abstract

Glyphosate (GP)-based herbicides have been widely applied to crops for weed control and pre-harvest desiccation. The objective of this research was to evaluate the effects of pre-harvest GP application on maize or how it physiologically alters this crop. Here, we applied four GP treatment (Control, GP150, GP200, and GP250) on maize lines of Z58 and PH6WC belonging to different maturity groups at grain-filling stages form DAP30 to DAP45. GP application significantly decreased the grain moisture content at harvest by 22–35% for Z58 and by 15–41% for PH6WC. However, the responses of grain weight to glyphosate vary with inbred lines and application time. A high concentration of glyphosate (GP250) reduced the grain weight of Z58 and low concentrations (GP150 and GP200) did not affect, while the grain weight of PH6WC significantly decreased under glyphosate treatment. In summary, our results revealed that timely and appropriate GP application lowers grain moisture content without causing seed yield and quality loss. GP application adversely affected photosynthesis by promoting maturation and leaf senescence. Meanwhile, it also enhanced non-structural carbohydrate (soluble sugars and starch) remobilization from the vegetative organs to the grains. Hence, GP treatment coordinates plant senescence and assimilate remobilization. RNA sequencing revealed that glyphosate regulated the transcript levels of sugar signaling-related genes and induced assimilate repartitioning in grains. This work indicates the practical significance of GP application for maize seed production and harvest, which highlights the contributions of source-sink communication to maize yield in response to external stress or pre-harvest desiccant application.

## Introduction

Maize (*Zea mays*) is cultivated worldwide and used for human food, animal feed, and bio-fuel. Grain moisture content is a limiting factor in machine kernel harvest (Wang and Li, 2017). Low grain moisture content at harvest reduces post-harvest grain drying costs and seed quality loss during storage. Pre-harvest desiccation economically and effectively enables timely harvest during unfavorable weather conditions. Glyphosate [N-(phosphonomethyl) glycine, GP] is an active ingredient in herbicides and is widely used for weed control (Orson and Davies, 2007). GP-based herbicides have been extensively used for pre-harvest desiccation of soybean (Whigham and Stoller, 1979; Pereira et al., 2015), rice (Bond and Bollich, 2007; He et al., 2015), beans (McNaughton et al., 2015; Parreira et al., 2015; Goffnett et al., 2016), wheat (Gélinas et al., 2018; Perboni et al., 2018; Malalgoda et al., 2019). This treatment has decreased seed production costs by drying crops and grains, promoting uniform maturation, and supporting timely combine harvesting (Xu et al., 2019). However, there are few studies on the responses of maize to GP application.

Glyphosate works by inhibiting 5-enolpyruvylshikimate-3-phosphate synthase (EPSPS) in the shikimate acid pathway, which ultimately prevents the plants subjected to glyphosate from synthesizing aromatic amino acids (Williams et al., 2000). Glyphosate acting as a non-selective herbicide adversely influences plant growth and may cause plant death (Geiger et al., 1986; Gomes et al., 2014). Nevertheless, previous studies on the effects of GP application on wheat, soybean and other crops suggested that appropriate GP treatment lowered the grains moisture content at harvest by promoting leaf senescence without any negative impact on crop yield (Whigham and Stoller, 1979; Perboni et al., 2018). The mechanisms by which glyphosate causes these changes in cereal grains are unknown. Moreover, glyphosate-induced damage to plants equates to external abiotic stresses (Kielak et al., 2011). In wheat, controlled water deficit treatments during post-anthesis may regulate the relationship between senescence and photosynthesis assimilates remobilization, accelerate grain-filling (Yang et al., 2001, 2004; Yang and Zhang, 2006). Maize stalk is a secondary sink that stores abundant carbohydrates during the vegetative stage. Remobilization of these reserves contributes to maize grain-filling (Kumar et al., 2019). Accelerating dehydration of maize kernels at harvest without diminishing crop yield has potential application value for the maize seed production. The present study aimed to determine whether the glyphosate used as pre-harvest desiccant on maize is feasible and to investigate the underlying mechanisms.

Here, we applied various glyphosate doses at grain-filling to evaluate the effects of glyphosate on maize. Our results demonstrated that timely and appropriate glyphosate effectively lowers moisture content at harvest without sacrificing crop yield and seed quality, which will be conducive to seed production and harvest. In addition, we disclosed that GP treatment coordinates plant senescence and assimilate remobilization by regulating sugar signaling in source-sink communication. These help clarify how GP application redistributes dry matter in the vegetative organs (sources) and the grains (sinks) of maize. This study also showed the practical significance of appropriate glyphosate in other staple cereal corps.

## Materials and Methods

### Materials and Glyphosate Treatment

The maize inbred lines Z58 and PH6WC, which are the parental lines of the most widely cultivated maize hybrid of Zhengdan958 (ZD958) and XY335 in China, respectively. Z58 and PH6WC belonging to GDD (growing degree days) maturity rating GDD2900 and GDD2650 (Wang, 1960) were used to investigate the effects of GP treatment on grain moisture contents and seed quality. The materials were grown at Experimental Station (36°9′N, 117°9′E) of Shandong Agricultural University during the growing season of June–September 2018. This region is a temperate continental monsoon climate with an average annual temperature of approximately 13°C, an average frost-free period of 195 days, and average annual precipitation of 697 mm, which occurs mainly from June to August. The soil type is a typical brown soil with a sandy loam texture and the winter wheat (*Triticum aestivum L*.) was the previous crop. The plowed soil (0–20 cm) contained 10.5 g kg^–1^ organic matter, 0.8 g kg^–1^ total N, 35.2 mg kg^–1^ readily available P, and 81.8 mg kg^–1^ readily available K. In the growth season, Nitrogen, phosphate, and potash fertilizers were applied as N 210 kg ha^–1^, P_2_O_5_ 75 kg ha^–1^, K_2_O 150 kg ha^–1^. The N fertilizer was applied at a ratio of 4:6 prior to sowing and at the ninth leaf (V9) growth stage, whereas the P and K fertilizers were applied only prior to sowing. The field-plot size was 4 m × 3.6 m with 6 lines (0.60 m between lines with the planting density of 67,500 plants/hm^2^, 120 plants included in every plot). The trials were conducted using the randomized block design with three field plots replications. All the plants were self-pollinated. The day of pollination was designated as 0 days after pollination (DAP0). We set four glyphosate treatments levels, Control (0 mg⋅L^–1^), GP150 (150 mg⋅L^–1^), GP200 (200 mg⋅L^–1^) and GP250 (250 mg⋅L^–1^), respectively. The glyphosate concentration used in this study is much lower than that of GP-based herbicide used in agriculture. Each treatment was applied at four maize developmental stages. 1.2 L of each GP solution was sprayed onto each plot by electric watering can. At maturity ten pollinated corns per plot and per treatment were harvested simultaneously at least three times to measure moisture content and grain weight. The widely cultivated stay-green maize hybrid Zhengdan958 (ZD958, GDD2800) was used in subsequent experiment to explore the effects of GP application on plant development, grain dehydration and the underlying mechanism involved. ZD958 was grown from June to September 2019. Meteorological characterization and agronomic management as described previously. The field-plot size was 10 m × 2.4 m and there were four lines (0.60 m between lines with the planting density of 67,500 plants/hm^2^, 200 plants included in every plot). Each plot was sprayed with 2 L GP solution. The GP solution consisted of ammonium glyphosate (Dehao, China. 88.8% saltwater-dispersible glyphosate ammonium granules; 80% active glyphosate) dissolved in water.

### Determination of Grain Moisture Contents, 100-Grain Weight and Seed Components

Sampled corns of Z58 and PH6WC were manually threshed after harvest. The moisture content of the GP-treated seeds was immediately measured at least three times with a handheld moisture meter for cereal (PM888, Kett, Japan). After the seeds were dried, the 100-grain weight was determined. The relative seed moisture, oil, protein, and starch levels were measured with a DA 7200 NIR (Perten, Stockholm, Sweden). The relative content of each component was multiplied by the 100-grain weight to calculate the absolute component content (g 100 grain^–1^).

### Seed Germination Test

The germination percentages of Z58 and PH6WC under GP-treatment were calculated according to the rules for seed testing (ISTA Rules for Seed Testing Association, 2014). The trials were conducted in sand with four 50-seed replicates per treatment for each maize line. The seedlings were allowed to grow for 7 days and the germination percentage was calculated. On the 7th day, all normal seedlings of each replicate were selected for the fresh weight. For recording dry weight, all normal seedlings were dried at 105°C for 30 min and then transferred to 80°C and dried until constant weight.

### Phenotype Analysis

At DAP35, GP200 was applied to the ZD958 plants. Plant and corns phenotypes were assessed at DAT10, DAT20, and DAT30 by randomly sampling separate maize plants. There were eight or ten field plots replicates, and each includes 2–3 individual plants. Leaf blades, sheaths, stems and ears of maize plant were separately harvested and weighted immediately. All the samples except the ears were heated to 105°C for 2 h and then to 85°C for 3 days until constant dry weight was obtained. All dried samples were weighted to determine the dry matter content (DM). They were then pulverized and used to evaluate the chemical component concentrations (mg⋅g^–1^ DM) in the various organs.

### Measurement of Non-structural Carbohydrates Content (NSC)

The dried samples of ZD958 were ground and their NSC (including soluble sugar and starch) content was determined based on anthrone-sulfuric acid method (Dubois et al., 1956). About 0.1 g powder were homogenized and extracted with deionized water. The supernatant was acidified, boiled, and treated with the anthrone reagent and the absorbance of the mixture was measured in a spectrophotometer (Biochrom S 2100) at 620 nm. Purified 100 μg mL^–1^ glucose (Sigma-Aldrich) was the calibration standard and sequential dilutions were prepared from it. The soluble sugar content was determined by interpolation from the glucose standard curve and expressed as mg⋅g^–1^ DM. The NSC content was measured three times. For the starch extraction, the aforementioned precipitate was boiled in deionized water and HClO_4_ was added with constant stirring. The mixture was centrifuged and the supernatant was used to determine the starch content by the same method as that described above for the soluble sugars.

### Measurement of Protein Content

All samples were dried and pulverized. The total N content in the leaves and stems was determined by a semimicro Kjeldahl method. Samples weighing 0.05–0.1 g were prepared and the total protein content was calculated using a Kjeldahl protein coefficient of 6.25. Soluble protein content was estimated by a Coomassie Brilliant Blue G-250 assay. About 0.15 g powder was homogenized and extracted with deionized water. Then 150 μL supernatant of each sample was pipetted into tubes, 1.5 mL Coomassie reagent was added, and the tubes were vortexed immediately. The mixtures were incubated at room temperature for 10 min and their absorbances were measured at 595 nm. The sample protein content was interpolated from a bovine serum albumin (BSA) standard curve.

### Determination of Structural Carbohydrates and Lignin

Structural carbohydrates and lignin were measured with an ANKOM 220 Fiber Analyzer (ANKOM Technol. Corp., Fairport, NY, United States) based on the method proposed by Goering and Van Soest (1970) and Van Soest et al. (1991). All samples were dried and pulverized. A filter bag was prepared and weighed. Its empty weight was designated M0 and 1 ± 0.1 g sample (M1) was added to it. The filter bag was washed with neutral detergent and dried to a constant weight (M2) at 105°C for ≥ 4 h. The filter bag was then washed with acid detergent, dried, and reweighed (M3). The filter bag was then immersed in 72% (v/v) H_2_SO_4_ for 3 h, rinsed with water, dried, and reweighed (M4). The content of hemicellulose, cellulose, and lignin was determined by the following procedure:

Hemicellulose⁢(g)=M3-M2;Cellulose⁢(g)=M4-M3;⁢Lignin⁢(g)=M4-M0.

The structural carbohydrate and lignin concentrations were expressed as mg⋅g^–1^ DM. Each component in every sample was measured at least twice.

### Determination of Glyphosate Residues on Grains

The GP residue content of the GP-treated seeds was measured based on national trade standard of China (SN/T 1923–2007) at inspection and quarantine technical center of Shandong Exit and Entry Inspection and Quarantine Bureau. 200 g seeds per treatment were used for testing.

### RNA-Seq Analysis

The RNA-seq data was obtained from ear-leaf blade (L) and sheath (Sh), and ear-bearing stem (St) of plants with or without GP-treated at DAT6 and DAT10, respectively. There were three replicates with each was sampled from five individual plants. RNA quantification and qualification were performed as described previously (Wen et al., 2017). Library construction and RNA sequencing were performed in Beijing Novogene Bioinformatics Technology Co., Ltd (Beijing, China). Clean data were obtained by removing reads containing adapter, reads containing ploy-N and low-quality reads from raw data. The clean reads were subsequently mapped to the B73 genome sequence^1^ using HISAT2 (Kim et al., 2015). The read numbers of each gene were counted by using HTSeq v0.6.1. And then FPKM of each gene was calculated based on the length of the gene and reads count mapped to this gene. FPKM, expected number of Fragments Per Kilobase of transcript sequence per Millions base pairs sequenced, The DESeq R package (1.18.0) was used for performing a differential expression analysis of the two groups (Wang et al., 2010). The resulting *P*-values were adjusted using Benjamini and Hochberg’s approach for controlling the false discovery rate. Genes with an adjusted *p* < 0.05 were considered differentially expressed genes (DEGs). GO and KEGG enrichment analyses of DEGs were implemented by the cluster Profiler R package (Yu et al., 2012). GO terms with corrected *P* < 0.05 were considered significantly enriched by DEGs. Similarly, the KEGG pathways with corrected *p* < 0.05 were assigned as significantly enriched pathways. The visualization of enrichment analysis was done on the ImageGP^2^.

### Statistical Analyses

For experiments where only two samples were compared (GP treatments and the control), an independent-samples *t*-test was used. For experiments involving more than two samples, statistical analysis was performed using ANOVA. One-way ANOVA followed with *Post Hoc* Duncan’s multiple comparisons test identified differences among treatment means. All statistical analyses were performed with SPSS 19.0 software (SPSS, Inc., Chicago, United States).

## Results

### Post-anthesis With GP Application Lowers Grain Moisture Content at Harvest

**FIGURE 1:**
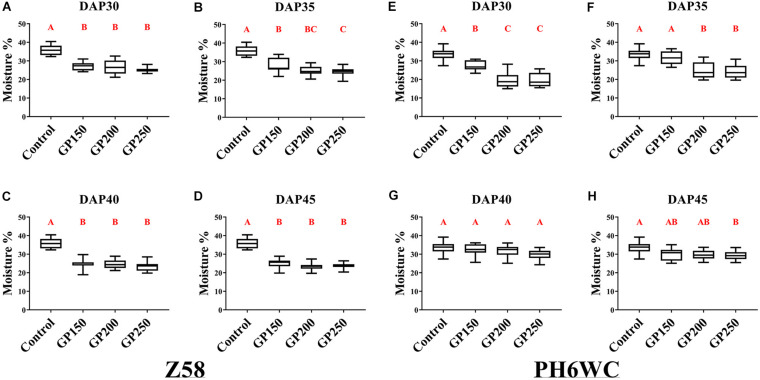
Effects of various GP concentrations and different GP application times on kernel moisture content at harvest. Kernel moisture content of Z58 **(A–D)** and PH6WC **(E–H)** under GP treatment at harvest. GP-treated (GP150, GP200, and GP250, which means 150, 200, and 250 mg L^–1^, respectively), and control maize were evaluated at DAP30 **(A,E)**, DAP35 **(B,F)**, DAP40 **(C,G)** and DAP45 **(D,H)** to assess effects of dehydration on seeds. There were three field plots replicates per treatment. For each field plot replicate, three or four technical replicates were carried out, and at least 10 individual corns were harvested for each technical replicate. Boxes indicate second and third quartile boundaries of data distributions. Whiskers indicate Q1 and Q3 values within 1.5× interquartile range. *N* ranged from 9 to 12 per treatment. Different letters denote significantly different treatment means within the same developmental stages at *P* < 0.01 according to one-way ANOVA followed with *Post Hoc* Duncan’s multiple comparisons test.

To examine the effects of GP on the grain dehydration, we initiated four GP treatments at 30 days after pollination (DAP30) and continued them at 5 days intervals until DAP45. All seeds were harvested at maturity, the time when the black layer has formed. The grain moisture content was then determined at harvest. Grain moisture content profiles of for Z58 and PH6WC under post-anthesis GP treatment are shown in Figure 1. Glyphosate reduced harvest grain moisture content to varying degrees. Compared with the control, the grain moisture in the treated seeds was significantly (*P* < 0.01) decreased by 22–35% for Z58 (Figures 1A–D) and by 15–41% for PH6WC (Figures 1E,F). Both lines presented with similar responses to the GP treatment gradients. The grain moisture content at harvest showed a decreasing tendency following treatment with increasing concentration of glyphosate.

Under normal growing conditions, the growth periods of Z58 (GDD 2900) was longer than that of PH6WC (GDD2650). The corn R5 stage (kernel dent stage, prior to R6 stage/physiological maturity, Nleya et al., 2016) vary for Z58 and PH6WC: at ∼DAP35-40 vs. ∼DAP30. Compared with Z58, the moisture content in the PH6WC grains had only minimally changed after the glyphosate was applied at the latter grain-filling stages (DAP40 and DAP45, Figures 1G,H). Hence, the responses of harvest grain moisture content to GP treatment vary with glyphosate rates and corn growth stages. Glyphosate is the active ingredient in certain herbicides and may have certain unknown effects on crop yield and/or seed quality. For this reason, grain weight, seed germination, and seedling growth of GP-treated maize were evaluated.

### Appropriate GP Application Minimally Influences Grain Weight and Does Not Reduce Seed Quality

**FIGURE 2:**
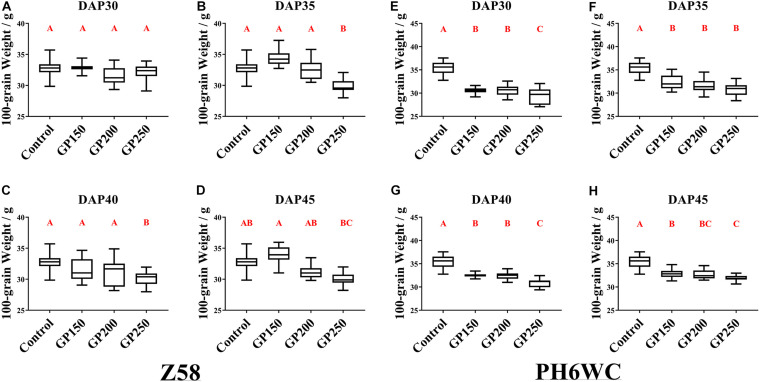
Effects of GP treatments on hundred-grain weight. Hundred-grain weight of Z58 **(A–D)** and PH6WC **(E–H)** under GP treatments at harvest. Plants were treated with glyphosate at DAP30 **(A,E)**, DAP35 **(B,F)**, DAP40 **(C,G)** and DAP45 **(D,H)**. There were three field plots replicates per treatment. For each field plot replicate, three or four technical replicates were carried out, and at least 10 individual corns were harvested for each technical replicate. Boxes indicate second and third quartile boundaries of data distributions. Whiskers indicate Q1 and Q3 values within 1.5× interquartile range. *N* range was 9–12 per treatment. Different letters denote significantly different treatment means within the same developmental stages at *P* < 0.01 according to one-way ANOVA followed with *Post Hoc* Duncan’s multiple comparisons test.

For Z58, there were no differences in 100-grain weight between the low concentration GP treatment (GP150, GP200) and the control. However, the GP250 treatment caused a 6.7–8.7% decline in the 100 grain weight relative to the control (Figures 2A–D). Figure 2 shows that Z58 and PH6WC responded differently to GP exposure. For PH6WC, the GP-induced decline in grain weight varied with treatment time and glyphosate rates (Figures 2E–H). The GP treatment significantly lowered the 100-grain weight by 7–16% and the decline increased with increasing glyphosate concentration (Figures 2E–H). Additionally, the decrease of grain weight became weaker gradually as the treatment time was closer to physiological maturity, with 13.6–16.6% decline at DAP30, 8.1–12.7% decline at DAP35 and DAP40, and 7–9.6% decline at DAP45. In summary, for relative stay-green Z58, appropriate GP application effectively dries maize grains but slightly increases yield loss. Although glyphosate had similar dehydration result on PH6WC, the 100-grain weight markedly decreased (Figures 1, 2).

We then investigated the effects of glyphosate on seed quality. A seed germination analysis disclosed that relative to the control, the germination rates for the seeds of Z58 and PH6WC subjected to glyphosate were essentially unchanged regardless of spraying time (Supplementary Figure S1). No significant differences were found between the control and GP treatments in terms of final germination percentage or seedling performance (Supplementary Figures S1, S2). Also, GP residue contents of GP-treated grains is less than 0.05 mg kg^–1^ grains, which is in line with relevant national standards of China (1 mg kg^–1^, GB 2763-2014).

Based on the results on the inbred lines, we comprehensively considered glyphosate effects on the grain dehydration rate and its impact on yield loss. Weather the effects of glyphosate on grains or the necessity of seed or crop production, GP application was more meaningful for stay-green or later maturing varieties by decreasing grain moisture for earlier or machine harvesting. What’s more, the practical applicability of glyphosate on maize hybrids remains to be clarified. Stay-green ZD958 (GDD 2800, belonging to later maturing groups) is similar to its maternal parent Z58 in terms of many agronomic traits. This widely cultivated maize hybrid in China, was selected to explore the effects of GP application on plant development, grain dehydration, and to clarify the underlying mechanism involved. In subsequent experiments, we applied GP200 at DAP35 to ensure effective grain dehydration at maturity without incurring yield loss.

### GP Application Coordinates Plant Senescence and Assimilate Remobilization

**FIGURE 3:**
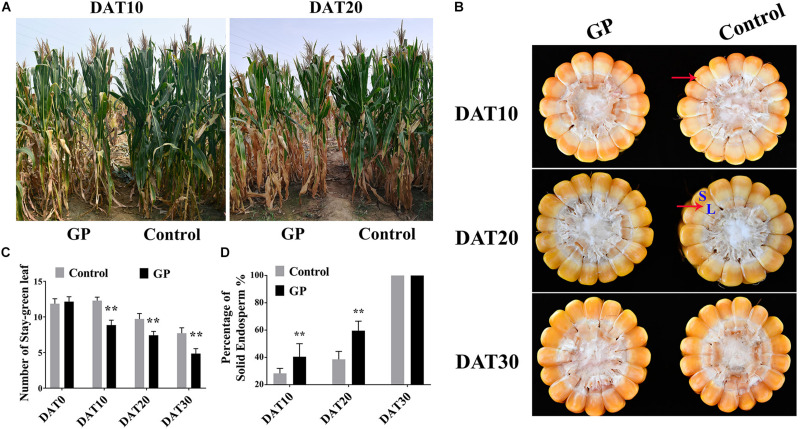
GP application at DAP35 regulated ZD958 senescence and grain-filling. **(A)** Phenotype of GP-treated and control ZD958 plants at DAT10 (10 days after treatment) and DAT20. **(B)** Cross-section of bottom half of corn cob at DAT10, DAT20, and DAT30. Red arrow indicates milk line position [boundary of solid (indicated by the blue letter, S) and liquid endosperm (L)]. **(C)** Relative number of stay-green leaves on control and GP-treated ZD958 plants at DAT0, DAT10, DAT20, and DAT30. There were eight or ten field plots replicates, and each includes 2–3 individual plants. Data are means ± *SD* (*n* = 30). **(D)** Relative kernel milk line progression between control and GP-treated maize. Milk line position represented by proportion of solid endosperm relative to whole kernel. Data are means ± *SD* (*n* = 30). ** indicate the significant difference between GP-treated and control at 1% levels using Student’s *t*-test, ns indicate non-significant.

The phenotypes of plant and corns subjected to GP200 were evaluated at DAT10 (10 days after treatment), DAT20 and DAT30, respectively. We established that at DAT10 and DAT20, glyphosate induced premature leaf senescence relative to that of the untreated control (Figure 3A). The GP-treated plant had fewer stay-green leaf number than the control and the difference between treatments was very large by DAT30 (Figure 3C). Leaf senescence is a fundamental developmental process related to the remobilization of carbohydrate in source-sink (Gan and Amasino, 1997; Song et al., 2016). We examined the phenotype of a corn cob cross-section after GP application to determine whether glyphosate influences grain morphology. Grain-filling progress is judged by the milk line of kernel (Afuakwa and Crookston, 1984) which is the boundary between the solid and liquid endosperm (Figure 3B, red arrows). The grain-filling rate increased with GP concentration (Figure 3B). The kernels of the GP-treated plants had more than twice the amount of solid endosperm as those from the untreated control plants (Figure 3D). We speculate that GP application might influence assimilate repartitioning in maize. Dry matter accumulation in the source and sink organs were quantified before and after GP application to elucidate the assimilate remobilization mechanism.

### GP Application Reduces Dry Matter Accumulation and Reallocates Assimilates Into Grains

**FIGURE 4:**
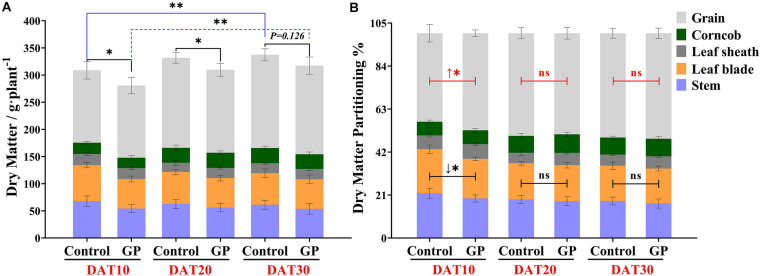
Effects of GP treatment on dry matter accumulation and partitioning in ZD958 maize organs. **(A)** Dry matter measured at DAT10, DAT20, and DAT30. Grain weight per plant was converted from wet grain weight. **(B)** Dry matter partitioning to stem, leaf, corncob, and sink (grains) after GP treatment. Black lines represent variance analysis of dry matter distribution in vegetative organs (stem and leaf) relative to untreated plants, and the red lines represent that of sink. There were eight or ten field plots replicates, and each includes 2–3 individual plants. Data are means ± *SD* (*n* = 8–10), * and ** indicate the significant difference at 5 and 1% levels according to Student’s *t*-test, respectively. ns indicate non-significant.

Ten to thirty days after GP treatment, the stems, leaf blades, leaf sheaths, and ears were sampled separately and their fresh weights were immediately measured. Relative to the control, there was a significant decrease (*P* < 0.01) in pre-plant fresh weight in the presence of glyphosate (Supplementary Figure S3). Supplementary Table 1A shows that GP application significantly decreased the total relative fresh weight mainly by reducing the fresh weight of the leaf blade and sheath and that of the ear by 21–33 and 8.7–11.7%, respectively, over the same period. To clarify the GP-induced source-sink carbohydrate remobilization, we measured the dry matte of vegetative organs (leaf and stem) and the grains. Figure 4A shows that total dry matter accumulation increased with maize development progress regardless of GP-treatment. Even at DAT30, the plants still presented with stay-green leaves (Figure 3C). In addition, GP application significantly reduced dry matter accumulation (Figure 4A and Supplementary Table 1B).

Previous research confirmed that the contributions of dry matter remobilization from stalk and the leaf were equally important for grain yield (Chen et al., 2014). It was reported that stem dry matter substantially contributes to wheat grain yield (Pheloung and Siddique, 1991). Here, we found that GP application altered dry matter allocation in vegetative organs and grains especially in the earlier phases after GP treatment (Figure 4B). The average dry matter partitioning to the grains was 47.4% at DAT10. This allocation was significantly higher than that measured for the control (43.2%) (Figure 4B). Glyphosate reduced the relative dry matter accumulation in the vegetative organs. For this reason, the entire plant dry matter content had decreased by DAT10 (Figure 4 and Supplementary Table 1B). However, dry matter partitioning was unchanged at DAT20 and DAT30 (Figure 4B). As maize development progressed and the effects of glyphosate declined, the difference between the GP treatments and the control in terms of grain DM had narrowed (Supplementary Table 1B). At the early stage (Phase I; DAT10–DAT20), glyphosate accelerated grain dry matter accumulation but had comparatively little impact by Phase II (DAT20–DAT30) (Supplementary Figure S4). As we had established that GP application influences source-sink communication, we investigated GP-induced changes in the chemical composition of the source and sink organs.

### Glyphosate Induced Changes in Maize Chemical Composition

**FIGURE 5:**
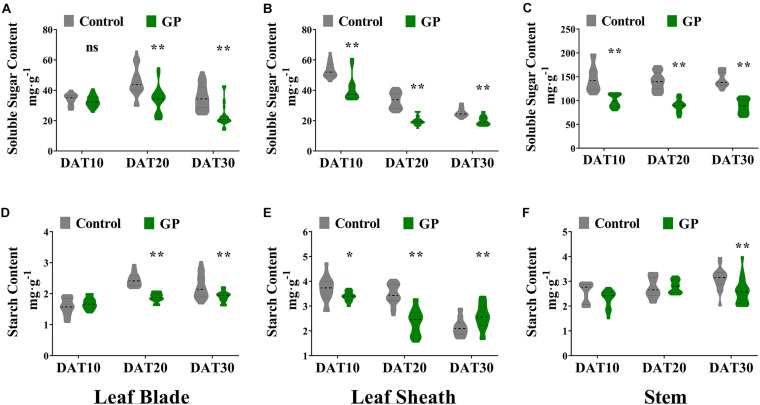
Effects of GP on NSC content in maize vegetative organs. Determination of soluble sugar and starch content in leaf blade **(A,D)**, leaf sheath **(B,E)**, and stem **(C,F)** 10–30 days after final treatment. Violin plots highlight the distribution density (gray or green part) of the soluble sugar contents. For each violin plot, thick dotted lines indicate median of data distribution while thinner dotted lines delimit boundaries of second and third quartiles. Upper and lower parts outside lines show 95% confidence intervals. There were eight or ten field plots replicates. The determination of NSC content was repeated three times. *N* ranged from 24 to 30 per treatment, obtained from 8 to 10 replicates. * and ** indicate the significant difference between GP-treated and control plants at 5 and 1% levels according to Student’s *t*-test, respectively. ns indicate non-significant.

We separately harvested the leaf, stem, and grain at DAT10, DAT20, and DAT30 and measured their NSC (non-structural carbohydrates such as soluble sugars and starch), structural carbohydrates (hemicellulose and cellulose), lignin, and protein. Relative to control, the soluble sugars contents were significantly (*p* < 0.01) lower in the leaves and stems of GP-treated plants at DAT10, DAT20, and DAT30 (Figure 5). Compared with the untreated, the average soluble sugar content in the leaf blades had declined by 3.8, 26, and 34% at DAT10, DAT20, and DAT30, respectively (Figure 5A). Relative to the control, the mean soluble sugar content in the leaf sheaths were 28, 40, and 21% lower at DAT10, DAT20, and DAT30, respectively (Figure 5B). Compared with the untreated, the average soluble sugar content in the stem had decreased by 32, 36, and 39% at DAT10, DAT20, and DAT30, respectively (Figure 5C). The soluble sugar content in the stem had fallen more than it did in the other vegetative organs. The relative DM content in the stem had declined by 46.8–56.5 mg g^–1^. The dramatic reductions in soluble sugar content induced by glyphosate indicated the major contribution of the stem to the final grain yield. The relative starch content decreased to varying degrees in the vegetative organs of GP-treated plants (Figures 5D–F). Following GP application, the NSC content had significantly declined compared with the control (Figure 5). The observed decrease in the NSC content of the vegetative organs closely paralleled the measured decrease in DM when glyphosate was applied post-anthesis (Figure 4A).

Overall, the relative structural carbohydrate levels in GP-treated organs were unchanged except for those in leaf blade which had significantly (*P* < 0.01) increased by 12.8–17.8%) (Supplementary Figure S5). The GP treatment did not alter total protein (total N) in the leaves or stems compared with the control (Supplementary Figures S5C,F,I). Glyphosate most dramatically changed the soluble sugar content in the vegetative organs relative to the control (Figure 5 and Supplementary Figure S5). Ongoing research is required to clarify GP-regulated relations of vegetative organs and grains. To this end, the impact of GP application on grain starch, protein, and oil content must be evaluated. In the present study, however, we detected no significant alterations in the absolute protein, oil, or starch (g 100 grain^–1^) content in GP-treated seeds compared with the control (g⋅100 grain^–1^) (Supplementary Figures S6A–C).

Previous studies reported that both vegetative N remobilization and N absorbed at silking and post-silking contributed to grain N (Chen et al., 2015; Have et al., 2017). Nevertheless, our results revealed that post-anthesis GP application had negligible effects on N remobilization from the vegetative organs to the grains at R5 or R6. Recent evidence indicates that post-silking N, P, K accumulation and these nutrition remobilization in maize are associated with the leaf senescence characters and planting density (Shao et al., 2020). Moreover, the seed starch content was ∼7 × greater than the seed protein content (Supplementary Figure S6). Under the GP treatments, NSC comprised most of the dry matter remobilized from the vegetative organs. NSC may be used to synthesize structural carbohydrates (Figure 5 and Supplementary Figure S5). Glyphosate reduced the total NSC content in the vegetative organs more than it raised their SC and lignin content. Glyphosate promotes NSC allocation to the grains via numerous metabolic processes. Therefore, we performed RNA-seq analysis to elucidate the mechanisms by which glyphosate remobilizes assimilates.

### Transcriptome Analysis of Maize Vegetative Organs in Response to GP Application

**FIGURE 6:**
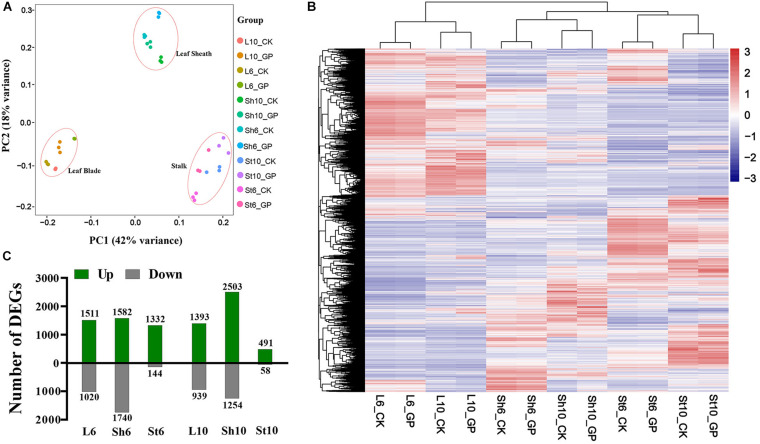
Effects of GP on maize at transcriptional level. **(A)** PCA of the transcriptomes of all samples. **(B)** Hierarchical clustering of vegetative organs with or without GP treatment. Clustering tree shows the variation of gene expression profiles within three biologicals and different organs. **(C)** DEGs in leaf blade (L), leaf sheath (Sh), and stem (St) between GP-treated and control at DAT6 and DAT10 of L6, L6_GP vs. L6_CK; Sh6, Sh6_GP vs. Sh6_CK; St6, St_GP vs. St_CK; L10, Sh10, and St10 are expressed in the same way.

GP-induced ear-leaf symptoms first appeared at 6 DAT6 and had worsened by DAT10. We acquired RNA-seq data for the ear-leaf blade (L), the sheath (Sh), and the ear-bearing stem (St) at DAT6 and DAT10. The 4.79 × 10^9^ clean reads that were generated were mapped to maize B73 reference genome (RefGen_V4, Jiao et al., 2017; Supplementary Datasheet 1). About 80% of reads were uniquely mapped (Supplementary Dataset 1) and used in FPKM-based quantitative gene expression analysis. Hierarchical clustering and principal component analysis (PCA) clearly discriminated the various groups (Figures 6A,B), suggesting that GP application may have organ-specific effects.

**FIGURE 7:**
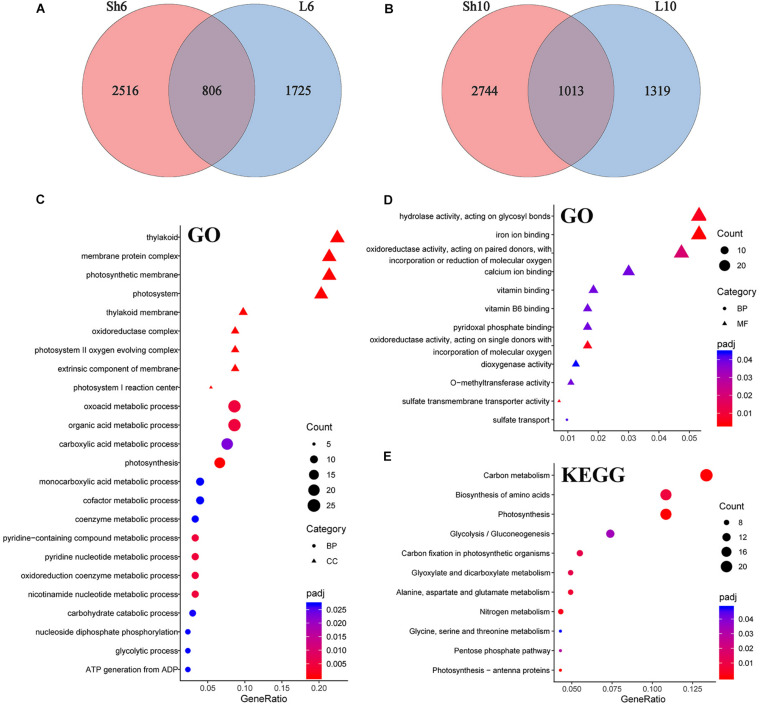
DEGs in leaf and their enrichment analysis. Veen diagram of unique and common DEGs in all samples at DAT6 **(A)** and DAT10 **(B)**. **(C,D)** GO analysis of DEGs common to leaf blade and sheath in **(A,B)**, respectively. **(E)** KEGG categories of common genes in leaf.

To characterize the effects of GP application on maize plants, differentially expressed genes (DEGs) were identified by comparing gene expression between the GP-applied plants and their respective controls (adjusted *P* < 0.05, fold change > 1.5). The raw reads were normalized and 24,241 genes were expressed (FPKM ≥ 1 for at least one sample) (Supplementary Datasheet 2). Of these, we detected 5,722 and 5,360 DEGs at DAT6 and DAT10, respectively (Figures 6C, Figures 7A,B and Supplementary Datasheet 3). To obtain information on the functions of DEGs, the Gene Ontology (GO) and Kyoto Encyclopedia of Genes and Genomes (KEGG) enrichment analysis were performed. At DAT6, GO enrichment of the common DEGs in the source organs (leaf blade and leaf sheath, Figure 7) identified several biological processes including photosynthesis, organic acid metabolic process, oxidoreduction coenzyme metabolic process, and carbohydrate catabolic process et al. (Figure 7C). All of the genes involved in photosynthesis were significantly downregulated in response to GP treatment (Supplementary Figure S7A). The GO molecular function analysis showed that at DAT10, the DEGs overlapping the leaf blade and the sheath were clustered in hydrolase activity, transporter activity, oxidoreductase activity et al. (Figure 7D). Most of DEGs (22/28) associated with hydrolase activity were glycosyl hydrolases (Supplementary Figure S7B). These are key components of carbohydrate metabolism that catalyze the hydrolysis of the glycosidic bonds in carbohydrates (cellulose, starch et al.) (Smith et al., 2005; Calderan-Rodrigues et al., 2018). The most significantly enriched KEGG pathways for the common genes are shown in Figure 7E. These included photosynthesis, carbon (C) or N metabolism, biosynthesis of amino acids, and carbon fixation et al.

**FIGURE 8:**
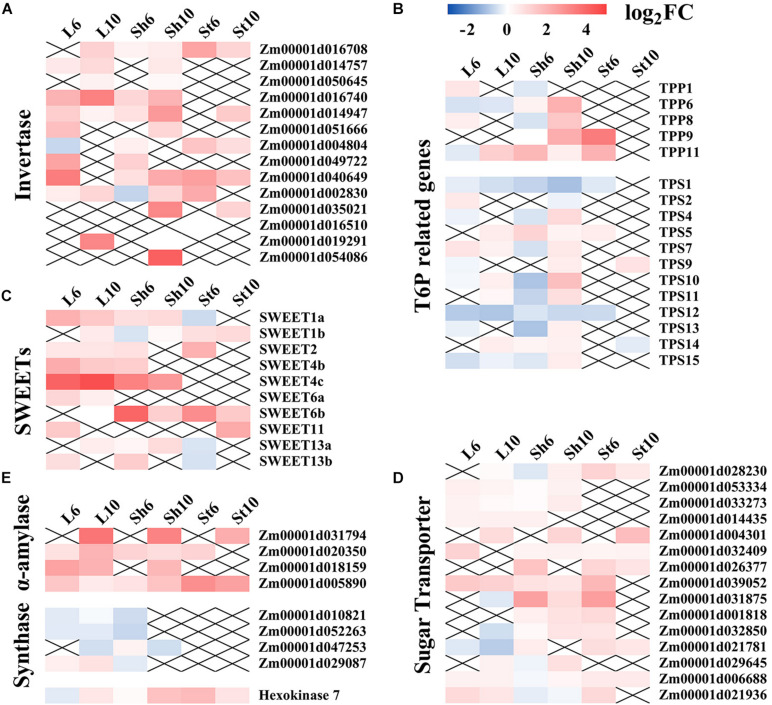
Expression levels of sugar-signaling related genes under GP treatment. Effects of GP on expression levels of invertase **(A)**, T6P (ttrehalose-6-phosphate) related genes **(B)**, SWEETs (sugars will eventually be exported by transporters) **(C)**, sugar transporters **(D)**, and other starch and sucrose metabolism genes **(E)**. TPP, T6P phosphatase. TPS, T6P synthase. X means there is no significant difference in expression level between GP-treated and control.

Enrichment analysis of the DEGs common to the leaf blade and the sheath soon after GP application identified several terms. Photosynthesis and its related genes were relatively downregulated and the genes involved in C and N metabolism were significantly altered with most upregulated under glyphosates (Figure 8 and Supplementary Figure S8).

The most significantly enriched GO terms in the stem related to oxidoreductase activity, lyase activity, transferase activity, and others (molecular function category) and several carbohydrate catabolism-related processes (biological processes category) (Supplementary Figure S9). The KEGG analysis uncovered the roles of glyphosate in amino acid biosynthesis and carbon metabolism-related pathways (Supplementary Figure S9) that are closely related to the remobilization of stored material from the stem to the grains.

## Discussion

### Versatility of Glyphosate on Crops

Glyphosate, as a non-selective herbicide, has a wide range of effects on various crop. It has been applied worldwide for pre-harvest desiccation of numerous crops like wheat, soybean, cowpea, etc. (Assis et al., 2019; Malalgoda et al., 2019). Our study confirmed the efficacy of glyphosate for pre-harvest maize desiccation (Figures 1, 3A). Here, GP-application realized a slightly higher relative protein content of ZD958 seeds (Supplementary Figure S6D). And the protein content in maize seed was significantly positively correlated with seed vigor (Wen et al., 2018). In general, glyphosate improved the quality of maize seeds, the similar result was from previous observations in wheat (Malalgoda et al., 2019). Both the pre-harvest management and enhanced seed quality, glyphosate showed a positive role on agricultural production.

Meanwhile, previous research revealed that glyphosate and its by-product aminomethylphosphonic acid (AMPA) adversely regulated plant physiological processes, such as photosynthesis or carbon metabolism, and caused oxidative stress and induced physio-biochemical changes in cereals (Servaites et al., 1987; Sergiev et al., 2006; Ahsan et al., 2008; Orcaray et al., 2012). For example, glyphosate induced leaf senescence, impaired seed germination, and lowered crop yield when it was applied prematurely to wheat (Perboni et al., 2018). Likewise, the effects of glyphosate on maize, such as accelerating senescence, decreasing dry matter, and lowering grain weight for some lines (Figures 2E–H, 3). From RNA-seq analysis, the molecular function terms enriched were involved in hydrolase activity and response to stress (Figure 7). Glyphosate treatment downregulated photosynthesis related genes and upregulated stress-responsive genes expression, like several nine-cis-epoxycarotenoid dioxygenases (NCED) (*Zm00001d007876*, *Zm00001d013689*, *Zm00001d018819*) (Supplementary Figures S7, S10B). Some vitamin B6 related genes also exhibited higher expression levels under GP treatment (Supplementary Figure S10A). Vitamin B6 has been identified as a potent antioxidant and responses to plant stress (Ehrenshaft et al., 1999; Chen and Xiong, 2005; Titiz et al., 2006). Our finding aligned with those of previous experiments and suggests that glyphosate adversely affects photosynthesis and other physiological processes.

How to coordinate the dual effects of glyphosate on crops, dosage effects may be one underlying mechanism. Interestingly, glyphosate works in a dose-dependent manner. While at low doses, GP stimulated photosynthesis in barley (Cedergreen and Olesen, 2010), CO_2_ assimilation in sugarcane (Nascentes et al., 2018), and seedling growth in maize (Velini et al., 2008; Brito et al., 2018). This effect is known as hormesis that any process in which a cell or organism exhibits a biphasic response to exposure to increasing amounts of a substance or condition. In general, low GP doses are stimulatory while high GP doses are inhibitory. Left unchecked, the latter may lead to plant death (Gomes et al., 2014). It is possible that oxidative damage might increase with glyphosate dose. However, relatively little is known about the mechanisms of the hormetic effects of glyphosate on plants.

Here for Z58, high concentration of glyphosate (GP250) reduced the grain weight, while low concentrations (GP150 and GP200) did not affect. However, the line of PH6WC exhibited contrasting behavior in response to GP application. Therefore, a major hindrance to the widespread use of glyphosate as a plant growth regulator is that its hormetic dose varies considerably and is difficult to control.

### Timely and Appropriate Application Optimizes Glyphosate Efficacy for Stay-Green Lines

Here, the responses of grain moisture content and grain weight to GP application vary with inbred lines, glyphosate concentration and treatment stage. Based on the days to physiological maturity, the lines of Z58 and PH6WC can be classified into early and late maturing groups. Z58 has a longer period silking-maturity than that of PH6WC. Study indicated remobilization of dry matter in maize as affected by maturity groups. The duration of period silking-maturity exhibited a positive correlation with dry matter accumulation and a negative correlation with mobilization of carbohydrates in vegetative organs (Pampana et al., 2009). For longer cycle stay-green maize germplasms (like Z58 and ZD958), photosynthesis continued until harvest and dry matter accumulation gradually increased. Thus, improving the remobilization of carbohydrates stored in vegetative organs for grain filling is the key to increase crop yield. The present study demonstrated that post-anthesis GP application might uniformly dry the maize plant and ear, facilitate grain dehydration (Figure 1, Supplementary Figure S3, and Supplementary Table 1), and then promote an earlier harvest. Meanwhile, glyphosate decreases dry matter by causing earlier leaf senescence which, in turn diminishes assimilate accumulation. Based on these results, the discrepancies among GP-induced leaf senescence, photosynthetic assimilate reduction, and crop yield stability may be explained by the fact that glyphosate enhances carbohydrate remobilization and grain-filling. However, GP application decreased the dry matter accumulation and did not significantly improve the mobilization of reserve carbohydrates in PH6WC. The reason needs to be determined with further analysis.

Our research focused on glyphosate as a pre-harvest maize desiccant used for crop production. In general, the grain moisture content at harvest decreased with increasing GP dose. Glyphosate concentration also markedly affected grain moisture and weight. GP250 most effectively dehydrated the grains at harvest (Figures 1, 2 and Supplementary Figures S1, S2). However, crop yield also decreased with increasing GP dose. Hence, we do not recommend the application of GP250 on maize.

Except for doses, the observed differences between Z58 and PH6WC in terms of their glyphosate responses could also be explained by their relative inconsistencies in kernel development. Pre-harvest desiccants are applied for harvest management. Therefore, the optimal application stage should be established in addition to the doses of desiccants. Researchers have attempted to assess the impact of pre-harvest GP application on crop yield and seed quality. When glyphosate was applied to desiccate soybean before physiological maturity, the yields were unaltered relative to the untreated control (Whigham and Stoller, 1979). In wheat, the negative effects of glyphosate on grain yield were minimized by applying the glyphosate as close to physiological maturity as possible (Perboni et al., 2018). For maize, our results suggest that appropriate stage for GP application is at R5 (kernel Dent Stage), prior to R6 (Physiological Maturity). The PH6WC dehydration rate at grain-filling is higher than that of the stay-green Z58. For this reason, the harvested PH6WC grain moisture content was insensitive to GP applied at DAP40 or DAP45 (close to R6 stage).

Considering all factors, our results showed that timely GP application may be necessary in order to minimize the negative impact of glyphosate for specific maize germplasms. Our results provide evidence for relative more-efficient GP application on maize. Therefore, both the dosage and the optimal plant development stage must be considered in the design of novel pre-harvest crop desiccants like glyphosate.

### Sugar Signaling in Source-Sink Communication Under GP Treatment

During grain-filling, appropriately GP application caused leaf senescence without diminishing grain weight. Leaf senescence may play a vital role in carbohydrate and nitrogenous nutrient recycling (N or carbohydrates) (Gan and Amasino, 1997). Non-structural carbohydrates and sugars participate in source-sink communication which, determines grain yield, nitrogen (N) uptake and transport and, ultimately, grain yield (Yu et al., 2015). As a rule, alterations in regulatory gene expression accompany the metabolic and physiological processes involved in source-sink communication (Yu et al., 2015).

GP could regulate key genes involved in starch and sucrose metabolism (Supplementary Figures S7, S8). Here, the effects of GP on carbon metabolism were also identified. Numerous studies have been conducted on the roles of sugars on the source-sink relationships that determine crop yield (Bihmidine et al., 2013; Ruan, 2014; Yu et al., 2015). Plant productivity increases with sink strength and/or source activity (Yu et al., 2015). During cereal crop grain-filling, sucrose is synthesized in the leaves and transported via the phloem to the grains (Ohshima et al., 1990). Nutrient remobilization from senescing leaves and stalk storage to the grains also determines the ultimate crop yield (Yu et al., 2015; Kumar et al., 2019). Sugar mobilization is also enhanced by sucrose transporter (SUT) activity or sugars will eventually be exported by transporters (SWEET) (Rolland et al., 2006; Ruan, 2014). An earlier study documented the roles of *ZmSWEET4c-*mediated hexose transport in maize seed filling (Nuccio et al., 2015). The function of *ZmSUT1* in maize leaves phloem loading has also been reported (Slewinski et al., 2009; Baker et al., 2016). Our RNA-seq analysis showed that glyphosate significantly upregulated *SUTs* and *SWEETs* (Figures 8C,D). GP application may enhance carbohydrate remobilization from the vegetative organs to the grains via numerous metabolic processes which are closely related to carbohydrate remobilization and yield stability. These findings help explain the contradiction between the observed decrease in dry matter and the lack of any significant decrease in grain weight under the GP treatments.

Invertase 1 and alpha-amylase catalytic related genes participate in sucrose hydrolysis and starch metabolism. A previous study reported that certain plant invertases regulate carbon partitioning during grain-filling (Yu et al., 2015). Here, GP upregulated several invertase genes and, by extension, regulated carbohydrate content (Figure 8A). We also found that glyphosate significantly enhanced the transcript levels of alpha-amylases (*Zm00001d031794*, *Zm00001d020350, Zm00001d018159*, *and Zm00001d005890*) which hydrolyzes alpha bonds in alpha-linked starch. However, GP downregulated starch and sucrose synthase genes (*Zm00001d010821*, *Zm00001d052263*, and *Zm00001d047253*) (Figure 8E). These results confirmed that GP application reduces the content of starch and certain soluble sugars (Figure 5). Glyphosate also controlled the transcription of sugar signaling-related genes and induced assimilate repartitioning into the grains. In this manner, photosynthetic assimilates could be partitioned or remobilized in source-sink organs.

It is now confirmed that SnRK1 (SNF1-related protein kinase 1) regulates source-sink relations by promoting nutrient mobilization and transport and influencing carbohydrate metabolism, starch biosynthesis, and senescence (Polge and Thomas, 2007; Yu et al., 2015; Wingler, 2018). T6P (trehalose-6-phosphate) inhibits SnRK1 and is considered a sucrose sensor (Figueroa and Lunn, 2016). Previous study established that *ZmTPP* (T6P phosphatase, which metabolizes T6P to trehalose) upregulation improved maize yield (Nuccio et al., 2015). Here, we demonstrated that GP regulated *TPP* and *TPS* (T6P synthase) expression (Figure 8B). Our transcriptional-levels data disclosed that GP-induced leaf senescence resembled natural senescence in that both processes remobilize nutrients from the aging leaves to the grains by regulating sugar signaling. Under the appropriate GP treatment, then, the plants will allocate relatively more assimilate to their sink.

## Conclusion

In conclusion, our results demonstrate that appropriate GP application lowers the grain moisture content by promoting maturity without compromising yield. We also conducted larger-scale field trials on maize hybrids. Glyphosate of 165–180 g active ingredient/hm2 could be applied at the R5 (Kernel Dent Stage), which facilitates the maize kernels dry enough to machine harvesting at physiological maturity. GP treatment coordinates plant senescence and non-structural carbohydrate reallocation from the vegetative organs to the grains by regulating sugar signaling in source-sink communications. Hence, this study showed the practical significance of GP application on maize seed production for the first time and highlighted the contribution of source-sink communication to crop yield especially in response to external stress. However, effects of glyphosate application on GP-based herbicide-tolerant maize, like genetically modified maize, were not discussed here. How the carbon remobilization is regulated by glyphosate needs further function analysis.

## Data Availability Statement

The original contributions presented in the study are publicly available. This data can be found here: NCBI, accession number: PRJNA646284.

## Author Contributions

CZ and LZ conceived the research, supervised the experiments, analyzed the data, and wrote the manuscript. LZ and LX performed most of the experiments. JH and YS performed the experiments of effects of GP application on maize. LX analyzed the RNA-sequencing data. All authors contributed to the article and approved the submitted version.

## Conflict of Interest

The authors declare that the research was conducted in the absence of any commercial or financial relationships that could be construed as a potential conflict of interest.

## Footnotes

1 ftp://ftp.ensemblgenomes.org/pub/plants/release-45/fasta/zea_mays/dna

2 http://www.ehbio.com/ImageGP/index.php/Home/Index/index.html

## Supplementary Material

Supplementary Figure 1 Effects of various GP concentrations and different GP application times on seed germination rate. Germination rate of Z58 **(A–D)** and PH6WC **(E–H)** under GP treatments at harvest. GP-treated (Control, GP150, GP200, and GP250) and control maize were evaluated 30–45 days after pollination to assess effects of GP on seed germination. Germination characteristics were recorded daily over a week period. Data are means ± *SD* (*n* = 3). There were three biological replicates and each included at least three technical replicates.

Supplementary Figure 2 Effects of GP treatments on seedling growth. Dry matter of Z58 seedlings **(A)** and fresh weight of PH6WC seedlings **(B)** at 7 days after imbibition were determined. Plants were treated with GP (Control, GP150, GP200, and GP250) from DAP30 to DAP45. Data are means ± *SD* (*n* = 3). There were three biological replicates and each includes at least three technical replicates.

Supplementary Figure 3 Fresh weight of stalk, leaf blade, leaf sheath, and era between GP-treated and control plants. Fresh weight was measured at DAT10 (10 days after GP treatment), DAT20 and DAT30. There were eight or ten field plots replicates, and each includes 2–3 individual plants. Data are means ± *SD* (*n* = 8–10), ^∗^ and ^∗∗^ indicate the significant difference at 5 and 1% levels according to Student’s *t*-test, respectively.

Supplementary Figure 4 Relative growth rate (RGR) in dry matter accumulation of seeds between GP-treated and control plants. Hundred-grain weight and moisture content were measured at DAT10, DAT20, and DAT30, the absolute dry weight (0% moisture) was converted from the wet weight (about 10–13% moisture). RGR was calculated from two consecutive harvests times. The phase I indicated the average growth rate from DAT10 to 20, and that of phase II was from DAT20 to 30. There were eight or ten field plots replicates, and each includes 2–3 individual plants. Data are means ± *SD* (*n* = 8–10). ^∗^ indicate the significant difference at 5% levels according to Student’s *t*-test.

Supplementary Figure 5 Effects of GP on structural carbohydrate, lignin, and total protein contents in maize vegetative organs. Determination of hemicellulose **(A)**, cellulose and lignin **(B)** and total protein **(C)** in leaf blade 10–30 days after final treatment. Contents of chemicals in leaf sheath **(D–F)** and stalk **(F–G)** were shown. Violin plots highlight the distribution density (gray or green part) of the contents. For each violin plot, thick dotted lines indicate median of data distribution while thinner dotted lines delimit boundaries of second and third quartiles. Upper and lower parts outside lines show 95% confidence intervals. There were eight or ten field plots replicates, and each includes 2–3 individual plants. *N* varied from 8 to 10 per treatment. ^∗^ and ^∗∗^ indicate the significant difference between GP-treated and control at 5 and 1% levels according to Student’s *t*-test, respectively.

Supplementary Figure 6 Effects of GP on the relative and absolute content of seed chemical composition. **(A–C)** The absolute protein, oil and starch contents per 100 grains. **(D–F)** The relative protein, oil, and starch content of seed. The seeds were harvested at 10 days after GP treatment (DAT10), DAT20 and DAT30. Multiply relative content of components by 100 grain weight to calculate the absolute content of components (g⋅100 grain^–1^). There were eight or ten field plots replicates, and each includes 2–3 individual plants. Data are means ± *SD* (*n* = 8–10), ^∗^ indicate the significant difference between GP-treated and control at 5% according to Student’s *t*-test. ns indicate non-significant.

Supplementary Figure 7 Expression pattern of DEGs associated with enriched GO terms photosynthesis **(A)** and hydrolase activity **(B)** in Figure 7.

Supplementary Figure 8 Expression pattern of DEGs associated with enriched KEGG pathways of carbon metabolism **(A)**, amino acid anabolism **(B)**, and nitrogen metabolism **(C)** in Figure 7.

Supplementary Figure 9 DEGs in stalk and their enrichment analysis. Veen diagram of unique and common DEGs in stalk at DAT6 and 10 **(A)**. GO analysis **(B)** and KEGG pathways **(C)** of common DEGs in **(A)**.

Supplementary Figure 10 Expression pattern of DEGs associated with enriched GO terms Vitamin B6 **(A)** and oxidareductase activity **(B)** in Figure 7.

Supplementary Table 1 Effects of GP treatment on dry matter content and fresh weight in ZD958 organs.

Supplementary Datasheet 1 Information of reads mapping to the reference genome.

Supplementary Datasheet 2 FPKM of expressed genes from three biological replicates of all samples.

Supplementary Datasheet 3 DEGs at DAT6 and DAT10 in GP-treated and control plant.
