# Supplementary material for: Proper Glyphosate Application at Post-anthesis Lowers Grain Moisture Content at Harvest and Reallocates Non-structural Carbohydrates in Maize
Source: Front Plant Sci. 2020 Dec 10;11:580883. doi: 10.3389/fpls.2020.580883 (PMC7758537; doi:10.3389/fpls.2020.580883)
Supplement: Supplementary Figure 1 — Effects of various GP concentrations and different GP application times on seed germination rate. Germination rate of Z58 (A–D) and PH6WC (E–H) under GP treatments at harvest. GP-treated (Control, GP150, GP200, and GP250) and control maize were evaluated 30–45 days after pollination to assess effects of GP on seed germination. Germination characteristics were recorded daily over a week period. Data are means ± SD (n = 3). There were three biological replicates and each included at least three technical replicates. [file Presentation_1.ZIP › Supplementary Material Presentation/Supplementary_Material.docx]

Supplementary Material

# Supplementary Data

Supplementary Figure S1. Effects of various GP concentrations and different GP application times on seed germination rate.

Supplementary Figure S2. Effects of GP treatments on seedling growth.

Supplementary Figure S3. Fresh weight of stem, leaf blade, leaf sheath and era between GP-treated and control plants.

Supplementary Figure S4. Relative growth rate (RGR) in dry matter accumulation of seeds between GP-treated and control plants.

Supplementary Figure S5. Effects of GP on structural carbohydrate, lignin and total protein contents in maize vegetative organs.

Supplementary Figure S6. Effects of GP on the relative and absolute content of seed chemical composition.

Supplementary Figure S7. Expression pattern of DEGs associated with enriched GO terms in Figure 7.

Supplementary Figure S8. Expression pattern of DEGs associated with enriched KEGG pathways in Figure 7.

Supplementary Figure S9. DEGs in stem and their enrichment analysis.

Supplementary Figure S10. Expression pattern of DEGs related to vitamin B6 and oxidareductase activity.

Supplementary Table 1. Effects of GP treatment on dry matter content and fresh weight in ZD958 organs.

Supplementary Datasheet 1. Information of reads mapping to the reference genome.

Supplementary Datasheet 2. FPKM of expressed genes from three biological replicates of all samples.

Supplementary Datasheet 3. DEGs at DAT6 and DAT10 in GP-treated and control plant.

# Supplementary Figures and Tables

## Supplementary Figures


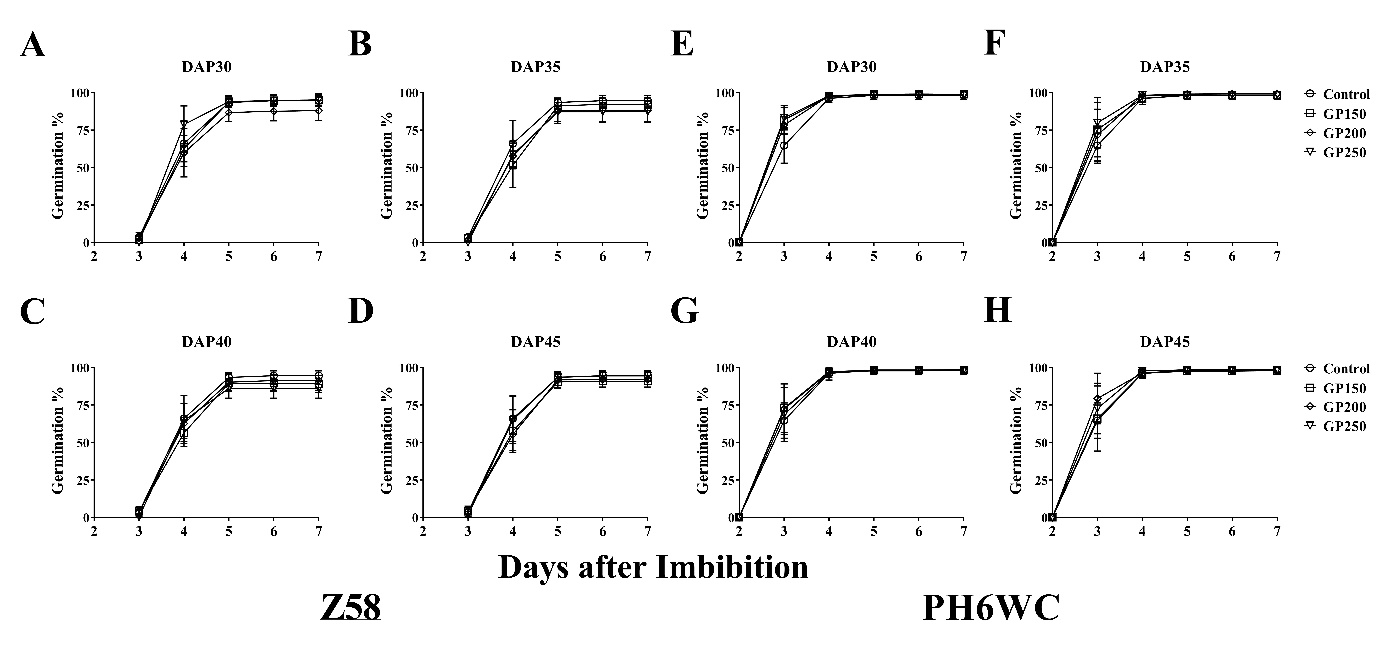


**Supplementary Figure S1.** Effects of various GP concentrations and different GP application times on seed germination rate. Germination rate of Z58 (A-D) and PH6WC (E-H) under GP treatments at harvest. GP-treated (Control, GP150, GP200 and GP250) and control maize were evaluated 30-45 d after pollination to assess effects of GP on seed germination. Germination characteristics were recorded daily over a week period. Data are means ± standard deviation (SD). There were three biological replicates and each included at least three technical replicates.


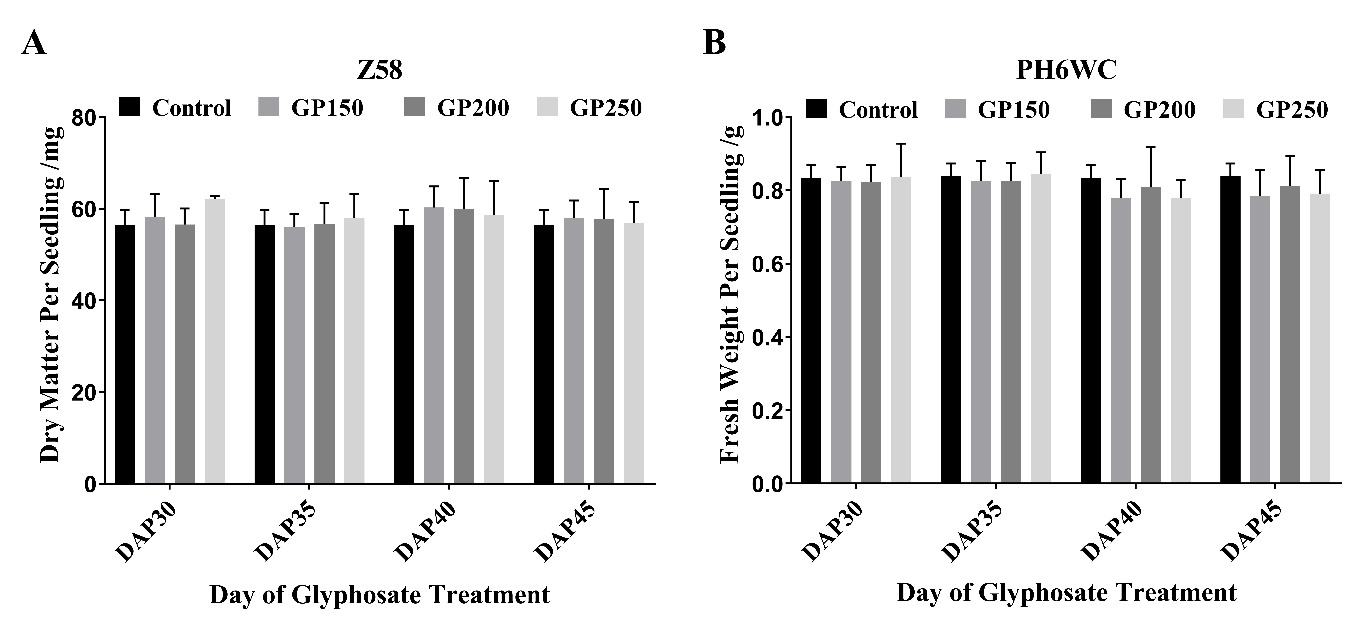


**Supplementary Figure S2.** Effects of GP treatments on seedling growth. Dry matter of Z58 seedlings (A) and fresh weight of PH6WC seedlings (B) at 7 days after imbibition were determined. Plants were treated with GP (Control, GP150, GP200 and GP250) from DAP30 to DAP45. Data are means ± SD. There were three biological replicates and each includes at least three technical replicates.

**
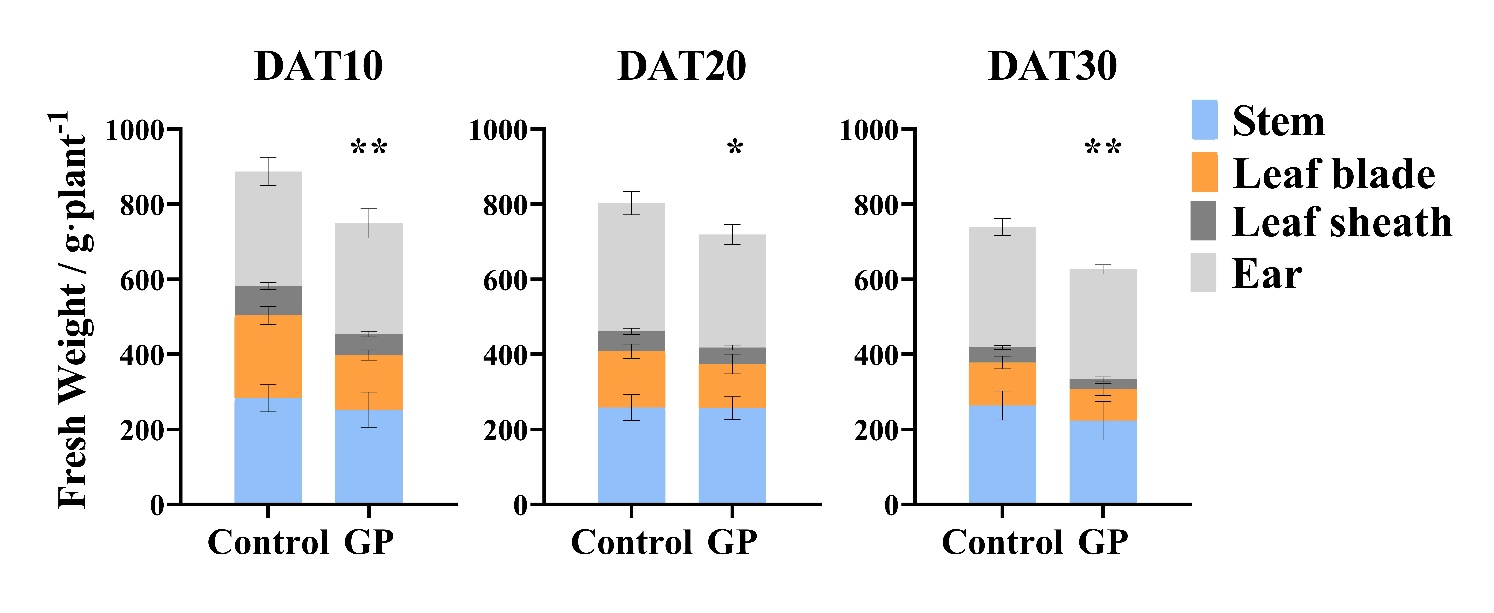
**

**Supplementary Figure S3.** Fresh weight of stem, leaf blade, leaf sheath and era between GP-treated and control plants. Fresh weight was measured at DAT10 (10 d after GP treatment), DAT20 and DAT30. Data are means ± SD (n=8 to 10), * and ** indicate the significant difference at 5% and 1% levels according to Student’s t test, respectively.


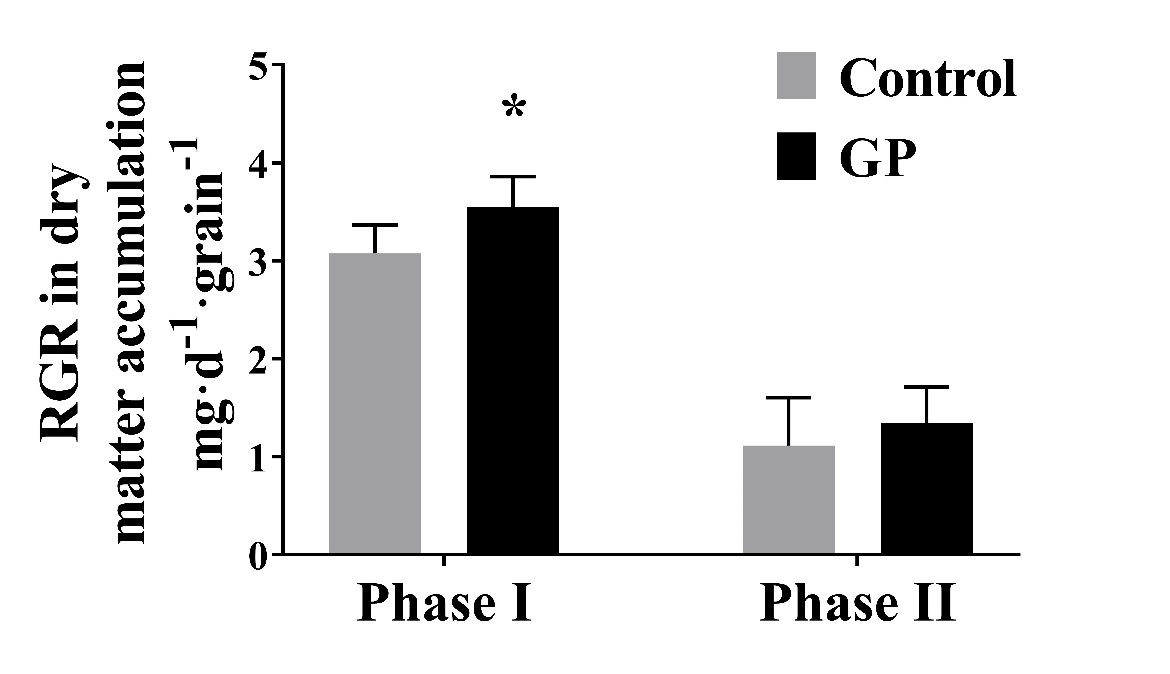


**Supplementary Figure S4.** Relative growth rate (RGR) in dry matter accumulation of seeds between GP-treated and control plants. Hundred-grain weight and moisture content were measured at DAT10, DAT20 and DAT30, the absolute dry weight (0% moisture) was converted from the wet weight (about 10-13% moisture). RGR was calculated from two consecutive harvests times. The phase I indicated the average growth rate from DAT10 to 20, and that of phase II was from DAT20 to 30. Data are means ± SD (n=8 to 10). * indicate the significant difference at 5% levels according to Student’s t test.

**
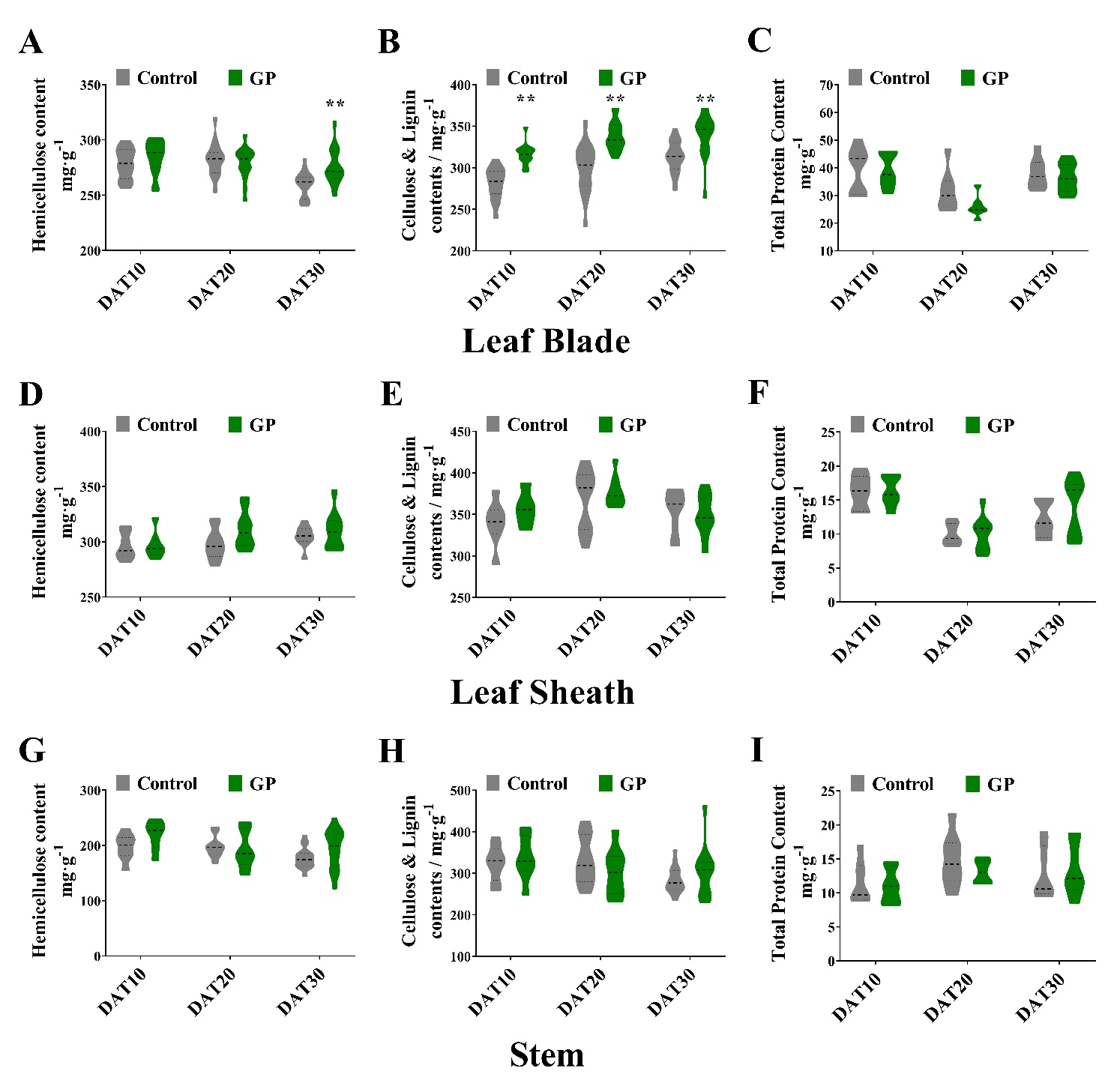
**

**Supplementary Figure S5.** Effects of GP on structural carbohydrate, lignin and total protein contents in maize vegetative organs. Determination of hemicellulose (A), cellulose & lignin (B) and total protein (C) in leaf blade 10-30 days after final treatment. Contents of chemicals in leaf sheath (D-F) and stem (F-G) were shown. Violin plots highlight the distribution density (gray or green part) of the contents. For each violin plot, thick dotted lines indicate median of data distribution while thinner dotted lines delimit boundaries of second and third quartiles. Upper and lower parts outside lines show 95% confidence intervals. N varied from 8-10 per treatment, * and ** indicate the significant difference between GP-treated and control at 5% and 1% levels according to Student’s t test, respectively.


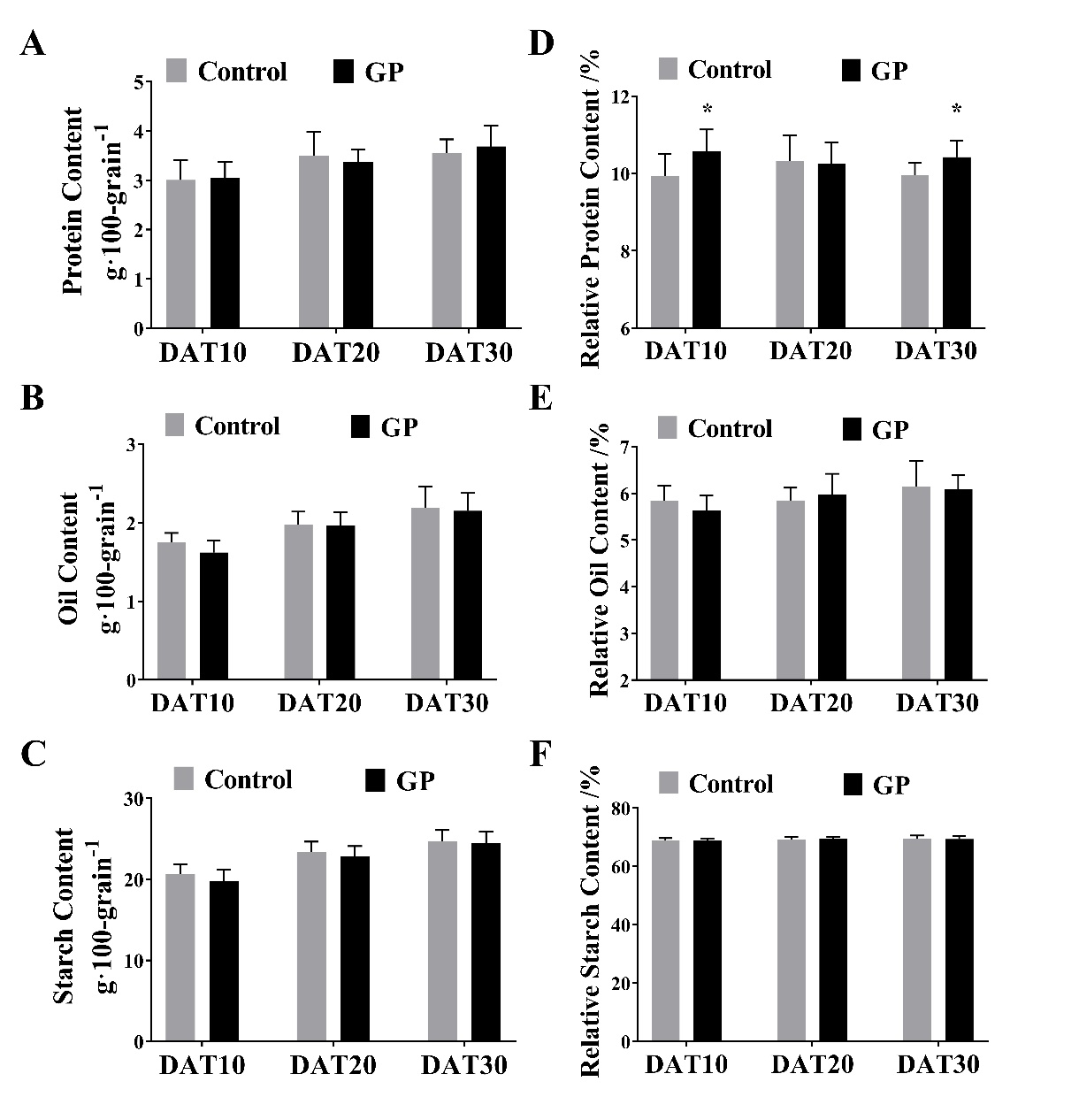


**Supplementary Figure S6.** Effects of GP on the relative and absolute content of seed chemical composition. A-C, the absolute protein, oil and starch contents per 100-grains. D-F, the relative protein, oil and starch content of seed. The seeds were harvested at 10 days after GP treatment (DAT10), DAT20 and DAT30. Multiply relative content of components by 100-grain weight to calculate the absolute content of components (g·100-grain-1). Data are means ± SD (n=8 to 10), * indicate the significant difference between GP-treated and control at 5% according to Student’s t test. ns indicate nonsignificant.


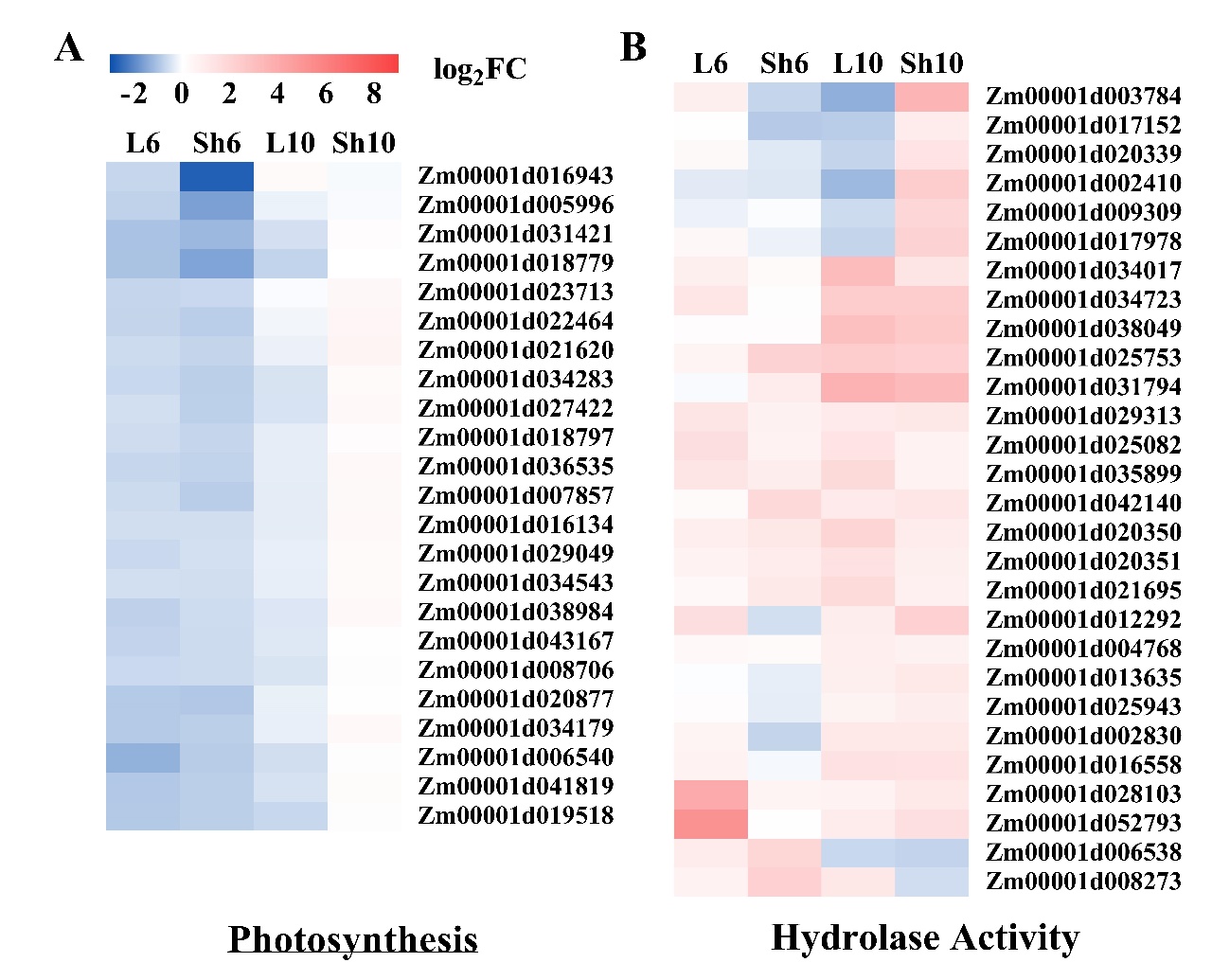


**Supplementary Figure S7.** Expression pattern of DEGs associated with enriched GO terms photosynthesis (A) and hydrolase activity (B) in Figure 7.


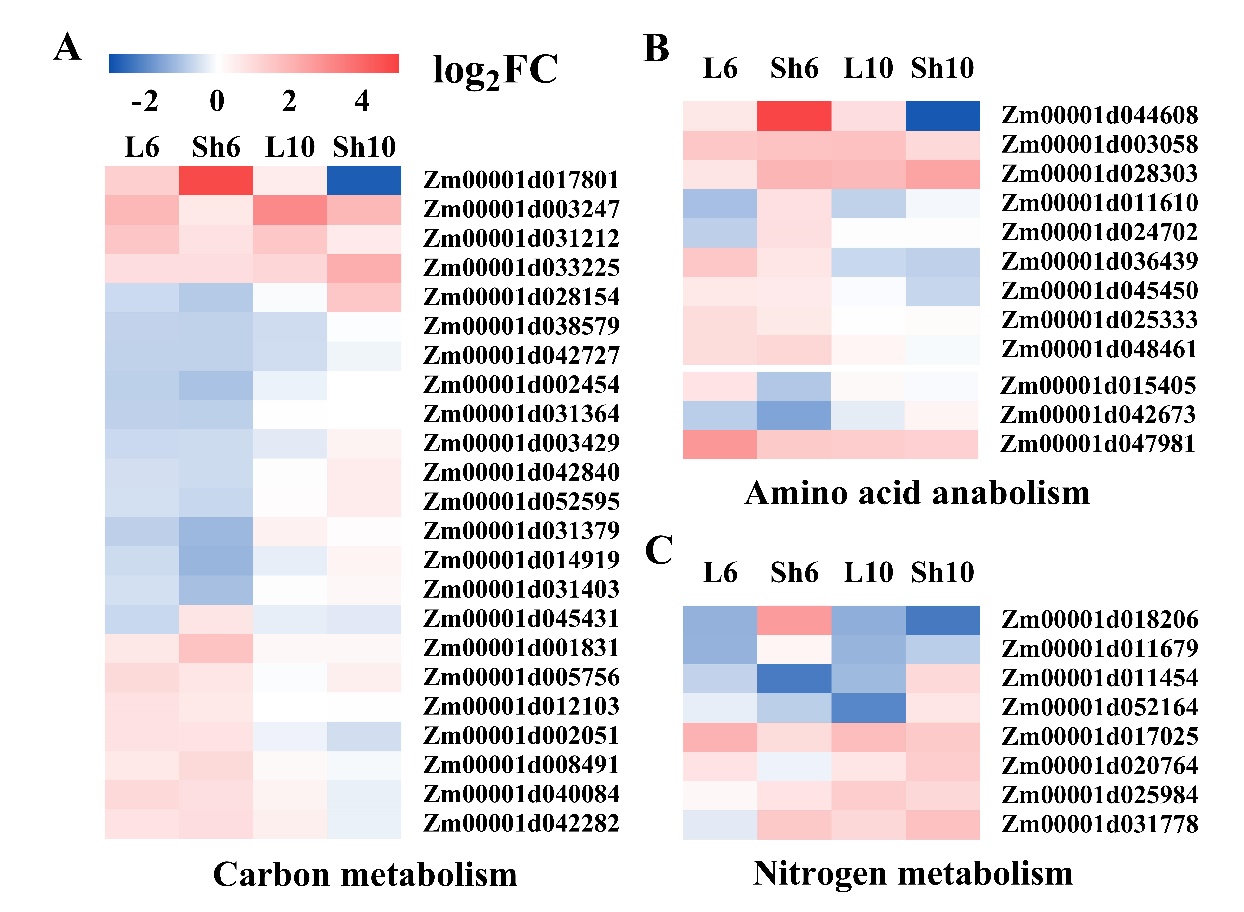


**Supplementary Figure S8.** Expression pattern of DEGs associated with enriched KEGG pathways of carbon metabolism (A), amino acid anabolism (B), and nitrogen metabolism (C) in Figure 7.


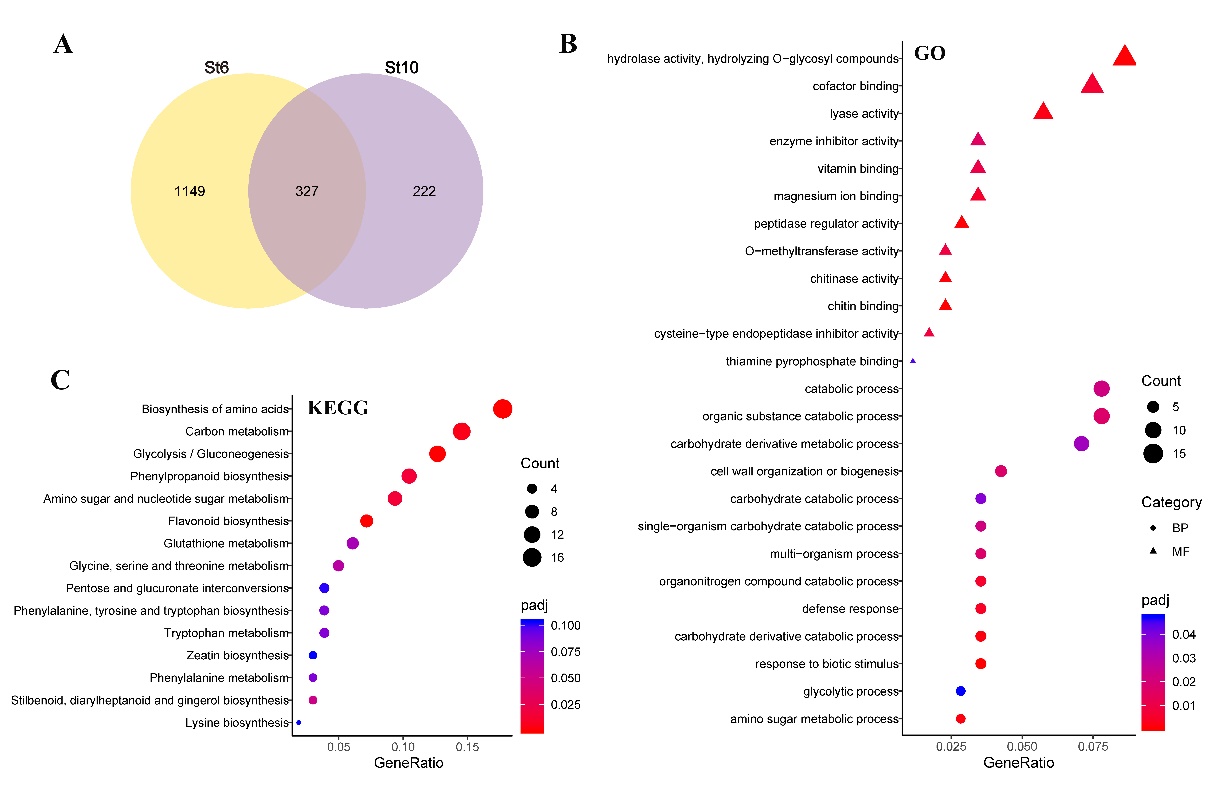


**Supplementary Figure S9.** DEGs in stem and their enrichment analysis. Veen diagram of unique and common DEGs in stem at DAT6 and 10 (A). GO analysis (B) and KEGG pathways (C) of common DEGs in (A).


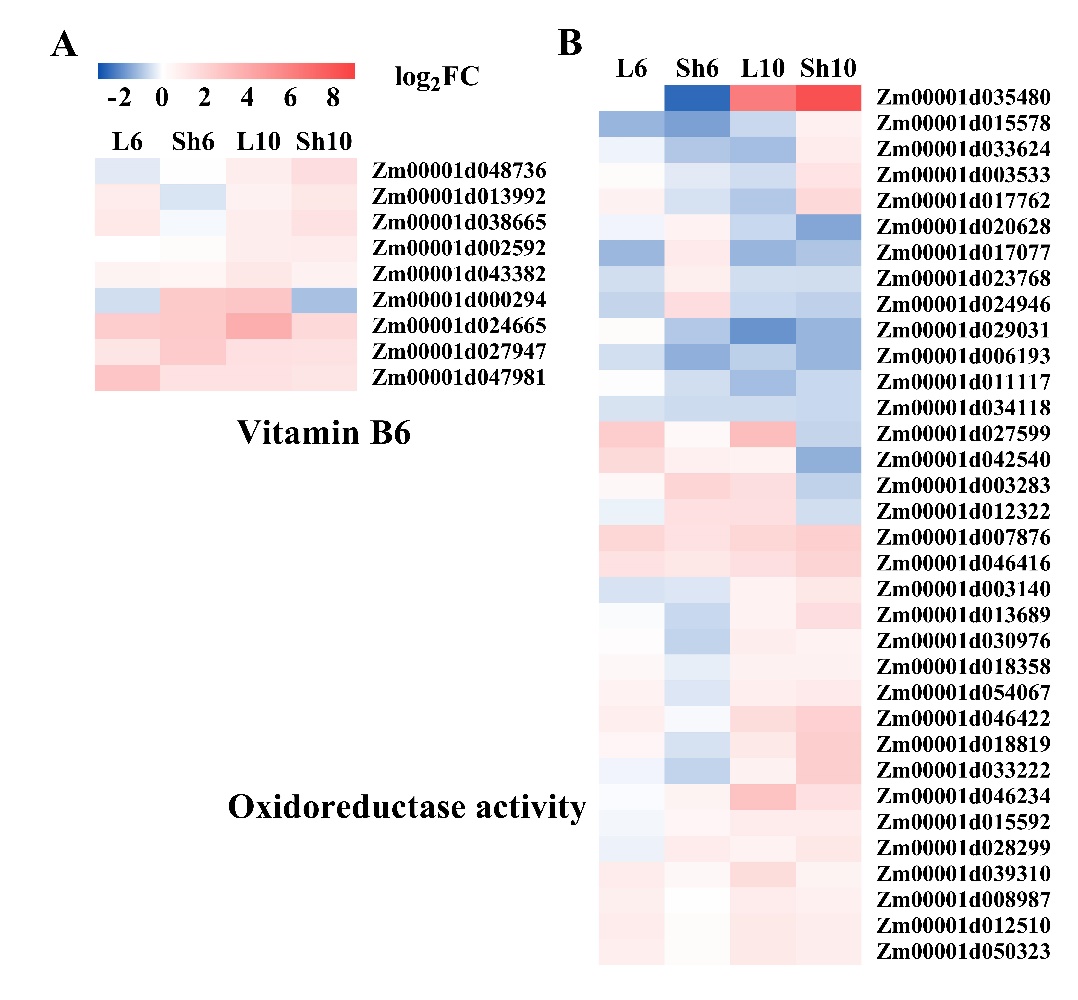


**Supplementary Figure S10.** Expression pattern of DEGs associated with enriched GO terms Vitamin B6 (A) and oxidareductase activity (B) in Figure 7.

## Supplementary Tables

Supplementary Table 1. Effects of GP treatment on dry matter content and fresh weight in ZD958 organs.

Supplementary Datasheet 1. Information of reads mapping to the reference genome.

Supplementary Datasheet 2. FPKM of expressed genes from three biological replicates of all samples.

Supplementary Datasheet 3. DEGs at DAT6 and DAT10 in GP-treated and control plant.
