# Supplementary figures and images for: Proper Glyphosate Application at Post-anthesis Lowers Grain Moisture Content at Harvest and Reallocates Non-structural Carbohydrates in Maize
Source: Front Plant Sci. 2020 Dec 10;11:580883. doi: 10.3389/fpls.2020.580883 (PMC7758537; doi:10.3389/fpls.2020.580883)

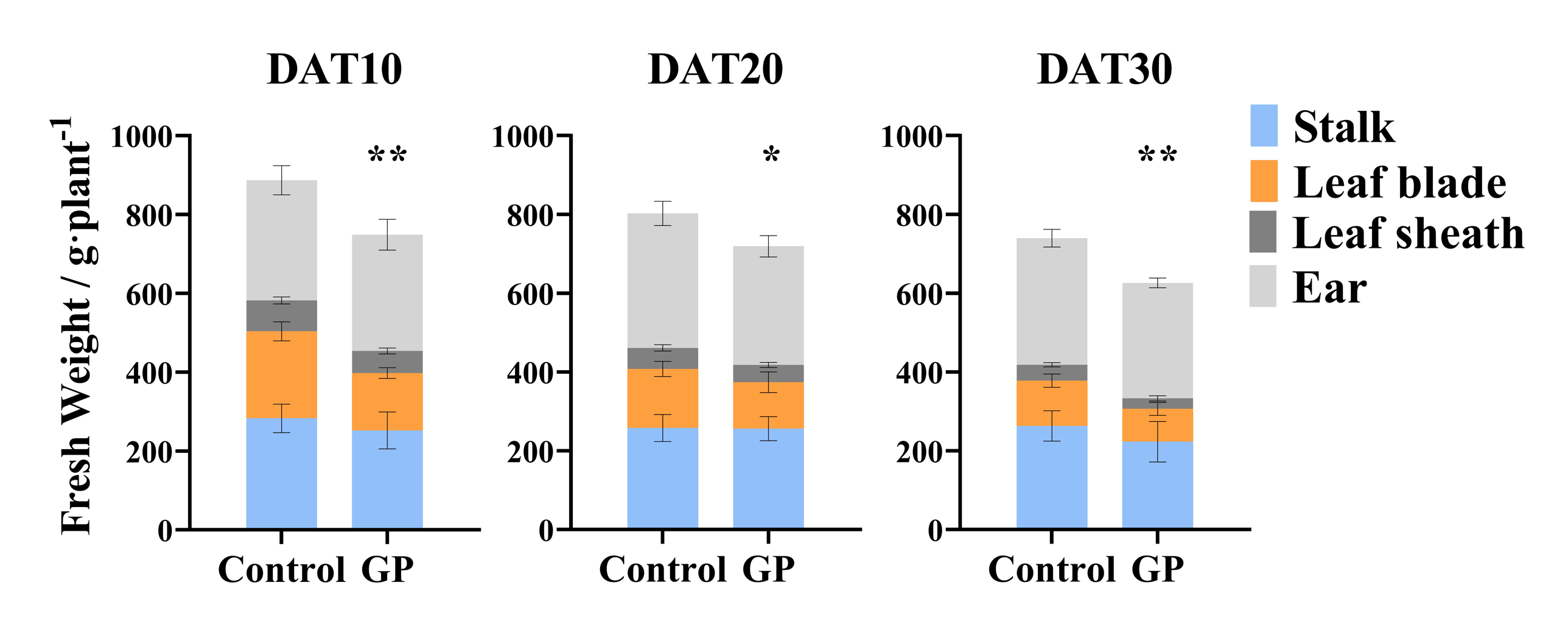

Supplement: Supplementary Figure 1 — Effects of various GP concentrations and different GP application times on seed germination rate. Germination rate of Z58 (A–D) and PH6WC (E–H) under GP treatments at harvest. GP-treated (Control, GP150, GP200, and GP250) and control maize were evaluated 30–45 days after pollination to assess effects of GP on seed germination. Germination characteristics were recorded daily over a week period. Data are means ± SD (n = 3). There were three biological replicates and each included at least three technical replicates. [file Presentation_1.ZIP › Supplementary Material Presentation/Figure S3.jpg]

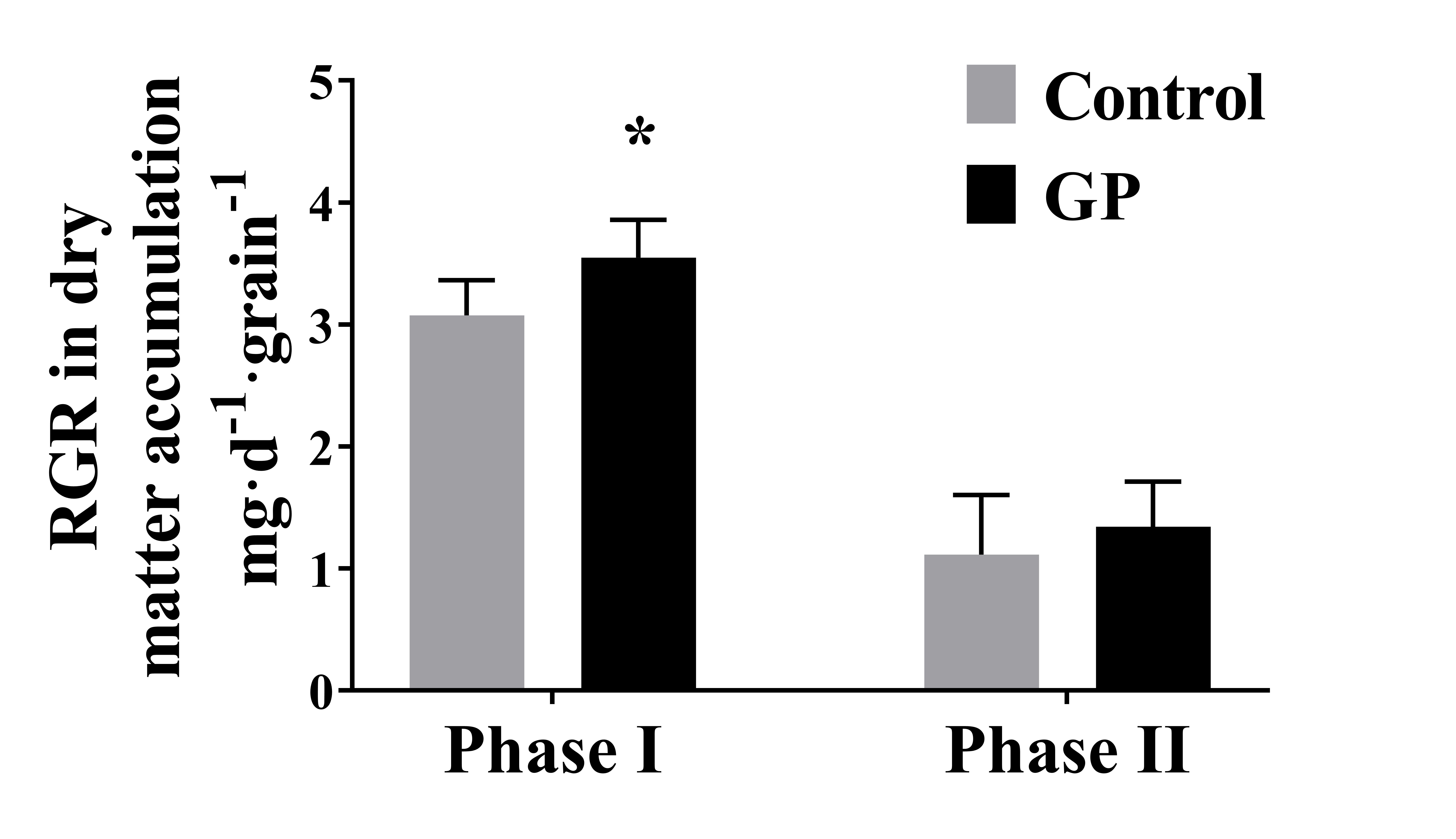

Supplement: Supplementary Figure 1 — Effects of various GP concentrations and different GP application times on seed germination rate. Germination rate of Z58 (A–D) and PH6WC (E–H) under GP treatments at harvest. GP-treated (Control, GP150, GP200, and GP250) and control maize were evaluated 30–45 days after pollination to assess effects of GP on seed germination. Germination characteristics were recorded daily over a week period. Data are means ± SD (n = 3). There were three biological replicates and each included at least three technical replicates. [file Presentation_1.ZIP › Supplementary Material Presentation/Figure S4.jpg]
